# Identification of quantitative trait loci for *in vitro* plant regeneration from leaf microexplants in cucumber (*Cucumis sativus* L.)

**DOI:** 10.1007/s13353-024-00927-3

**Published:** 2024-12-23

**Authors:** Renata Słomnicka, Magdalena Cieplak, Magda Antosiewicz, Alicja Sadłos, Aleksandra Galczak, Karolina Kaźmińska, Grzegorz Bartoszewski

**Affiliations:** Department of Plant Genetics Breeding and Biotechnology, Institute of Biology, Warsaw University of Life Sciences, SGGW, Warsaw, Poland

**Keywords:** *Cucumis sativus* L., *In vitro* plant regeneration, Organogenesis, QTL mapping, Recombinant inbred lines (RILs), Transgression

## Abstract

Plant regeneration in tissue cultures is crucial for the application of biotechnological methods to plant breeding. However, the genetic basis of *in vitro* plant regeneration is not fully understood. For cucumber, regeneration protocols from different types of explants have been reported, but thus far, the molecular basis of regeneration from cotyledon explants has only been studied. The aim of this work was to identify quantitative trait loci (QTLs) for *in vitro* plant regeneration from cucumber leaf microexplants. Plant regeneration was evaluated using a population of recombinant inbred lines (RILs) developed from a cross between line B10, characterized by high regeneration efficiency, and the low regeneration efficiency line Gy14. All RILs were scored for frequency of callus formation, organogenesis, and shoot regeneration. RILs with regeneration efficiencies higher than that of line B10 have been observed. QTLs for the frequency of organogenesis and shoot regeneration were identified. All the QTLs were mapped on cucumber chromosome 6, explaining 11.9 to 20% of the phenotypic variance. The major-effect QTL for organogenesis *or6.1* was located on the upper arm of chromosome 6. The QTLs for shoot regeneration frequency, *sr6.1A* and *sr6.1B*, were located on the lower arm of chromosome 6. Analysis of the genomic region corresponding to these QTLs combined with gene expression profiling revealed that *CsARF6* and *CsWOX9* are gene candidates underlying these QTLs. This study is a step toward identifying the genes controlling the ability of cucumber plant regeneration from leaf explants.

**Supplementary Information:**

The online version contains supplementary material available at 10.1007/s13353-024-00927-3.

## Introduction

Cucumber (*Cucumis sativus* L.) is an economically important vegetable crop. It is also a model plant species for studying the genetics of sex determination, vascular development, organellar transmission, fruit morphology, and other traits unique to cucurbits (Luo et al. 2023; Park et al. 2021; Grumet et al. 2023). Recent progress in cucumber genomics has contributed greatly to the identification of genes and quantitative trait loci (QTLs) for cucumber improvement (Feng et al. 2020; Wang et al. 2020; Liu et al. 2021; Zhang et al. 2021; Wang et al. 2022a, 2022b).

The ability to regenerate plants from somatic cells or protoplasts was described at the beginning of the twenty-first century. Since that time, many *in vitro* regeneration systems for different plant species have been established. These protocols are essential for experimental biology, plant breeding, agricultural biotechnology, and plant *in vitro* propagation (Thorpe 2007). Plants can be regenerated *in vitro* through organogenesis or somatic embryogenesis, directly from an explant or indirectly from a callus (Evans et al. 1981). In cucumber, several *de novo* plant regeneration protocols have been established, including direct and indirect regeneration via organogenesis or somatic embryogenesis (Tan et al. 2022). One of the regeneration protocols based on organogenesis from leaf explants was described by Burza and Malepszy (1995). This protocol takes advantage of small leaf microexplants (2–4 mm^2^) that are easy to collect in large amounts from a single leaf. It has been successfully utilized in cucumber genetic studies and biotechnology for doubled haploid production, cucumber transformation, regeneration of virus-free cucumber plants, and studying the mitochondrial genome (Gałązka et al. 2015; Szwacka et al. 2002; Yin et al. 2004; Mróz et al. 2015; Holz et al. 2019; Del Valle-Echevarria et al. 2015).

Plant cell and tissue cultures are widely used in modern agriculture, and there has been progress in understanding the cellular and molecular mechanisms underlying plant regeneration abilities (Xu and Huang 2014; Chen et al. 2024). In cucumber, several attempts have been made to identify genes controlling *in vitro* plant regeneration. Nadolska-Orczyk and Malepszy (1989), based on genetic analysis, reported that three independent genes characterized by complementary and additive interactions control cucumber plant regeneration from leaf explants. Wang et al. (2018) used QTL mapping and a genome-wide association study (GWAS) to identify cucumber loci responsible for direct regeneration from cotyledon explants. They identified QTLs for shoot regeneration from cotyledon explants on chromosomes 1, 3, and 6, with a major-effect QTL on chromosome 1. Further investigation allowed identifying a gene encoding an ortholog of the *Arabidopsis* J3 protein regulating PM H^+^-ATPase activity (Csa1G642540), as a candidate gene for shoot regeneration from cotyledonary explants (Wang et al. 2018). Moreover, several genes differentially expressed during cucumber somatic embryogenesis were identified, namely, *CsCUS1* and *CsSCR*, which encode MADS-box and SCARECROW transcription factors; *CsXTH1* and *CsXTH3*, which encode xyloglucan endotransglucosylases/hydrolases; and *CsSEF1*, which encodes a putative zinc finger protein (Filipecki et al. 1997; Malinowski et al. 2004; Grabowska et al. 2009; Wiśniewska et al. 2012 and 2013).

The aim of this study was to identify QTLs controlling highly efficient *in vitro* plant regeneration from cucumber leaf explants. To identify these QTLs, a population of recombinant inbred lines (RILs) characterized by opposite regeneration efficiencies was used. The RILs were tested under *in vitro* conditions, and QTLs for indirect regeneration efficiency from leaf microexplants were identified. The candidate genes underlying major QTLs for regeneration efficiency are discussed.

## Materials and methods

### Plant material

The mapping population was developed by crossing two highly inbred cucumber lines, Gy14 and B10, which are characterized by opposite *in vitro* plant regeneration efficiencies. Gy14 is characterized by low regeneration efficiency and was developed in the USA as a gynoecious line with white-spined fruits. The B10 line is characterized by high regeneration efficiency and was developed from the Central European cultivar “Borszczagowski”, a monoecious line with black-spined fruits. The recombinant inbred lines were generated by single-seed descent of greenhouse growth plants at the Wolica Experimental Station of the Department of Plant Genetics Breeding and Biotechnology at Warsaw University of Life Sciences (Poland). This population was described in our previous study (Słomnicka et al. 2018).

### *In vitro* regeneration from cucumber leaf microexplants

The parental lines Gy14 and B10 as well as the F_1_ and 92 F_8_ RILs were used to assess plant regeneration from leaf microexplants. A regeneration protocol developed by Burza and Malepszy (1995) with minor modifications was used (Supplementary Fig. S1). Cucumber seeds were surface-sterilized in 75% (v/v) ethyl alcohol for 60 s followed by 20% commercial bleach for 20 min and rinsed three times with sterile water. After seed coat removal, the seeds were placed in 0.4-L glass jars filled with 50 mL of half-strength MS medium (Murashige and Skoog 1962) supplemented with 10 g·dm^−3^ sucrose and 7.5 g·dm^−3^ Microagar (Duchefa, Haarlem, The Netherlands). Plants were grown under growth chamber conditions (25 °C, 16-h photoperiod, 40–50 μmol·m^−2^·s^−1^ light) for 14 days. The first or second true leaf from the apical meristem was collected and cut into small pieces (2–4 mm^2^) using commercial scalpel blades. These microexplants were placed on induction 1.7 medium supplemented with 1 mg·dm^−3^ 2,4-D (2,4-dichlorophenoxyacetic acid) and 1 mg·dm^−3^ 2-iP (N6-(delta 2-isopentyl)adenine) and incubated in the dark at 25 °C for 2 weeks. Next, the explants were transferred to hormone-free 1/6 medium and incubated at 25 °C with light (40–50 μmol·m^−2^·s^−1^) for 16 h and in the dark for 8 h. After every 2 weeks, the explants were transferred to fresh 1/6 medium (Supplementary Fig. S1). Two independent regeneration experiments were performed. In each experiment, four to six replicates of each line were used. Two weeks after the initiation of *in vitro* culture, the frequency of callus formation (CF) was evaluated as the percentage of explants that formed calli among all replicates. After 10 weeks, the frequency of regeneration was scored from all replicates and expressed as the percentage of explants regenerating either organs such as roots, leaf-like structures, buds or shoots (organ regeneration frequency, OR), or only shoots (shoot regeneration frequency, SR).

### Statistical analysis

The mean regeneration frequency of the replicates was subjected to statistical analysis. Basic statistics such as the mean, standard deviation (SD), and frequency distribution of regeneration among the RILs were calculated using GraphPad Prism v8.0 (GraphPad Software Inc., San Diego, CA, USA). To assess the effect of genotype on regeneration efficiency, data from the genetically uniform lines were subjected to a unifactorial analysis of variance (one-way ANOVA), and the means for the different traits were separated by a Tukey post hoc test (*p* < 0.01) using Statistica 13.1 (StatSoft, Tulsa, OK, USA). The normality of the distribution of the phenotypic data was tested by the Shapiro–Wilk test.

### Identification of QTLs for *in vitro* regeneration

The genetic map including DArTSeq and simple sequence repeat (SSR) markers and the QTL mapping approach were described previously by Słomnicka et al. (2018). Interval mapping (IM) for QTL detection was conducted with MapQTL 5.0 software (Van Ooijen 2004). The possibility of QTL existence was scanned on every chromosome at intervals of 1 cM. Genome-wide LOD thresholds (*p* ≤ 0.01) were empirically determined for the trait using the permutation test (PT) of MapQTL with 1,000 iterations. Based on the permutation tests, a threshold logarithm of odds (LOD) value of 3.1 (α = 0.01) was used to determine the presence of a QTL. Each locus was named by an abbreviation of the trait followed by the chromosome number and locus number. Genomic regions corresponding to each QTL were defined using the cucumber B10 v3 genome (Osipowski et al. 2020) supported by Gy14 v2.1 genome to determine chromosomal affiliation of linkage groups (Yu et al. 2023).

### Development of CAPS and INDEL Markers

To increase the density of the genetic map within the lower arm of chromosome 6, the genome sequences of B10 v3 and Gy14 v2.1 available at Cucurbit Genomics Database v2 (CuGenDBv2) (Yu et al. 2023) were aligned, and polymorphisms were identified. Single-nucleotide polymorphisms (SNPs) or insertion-deletions (INDELs) were selected for cleaved amplified polymorphic sequences (CAPS) or INDEL marker development (Supplementary Table S1). Primers were designed using Primer 3 v4.1.0 software (Untergasser et al. 2012) and synthesized by Genomed S.A. (Genomed SA, Warsaw, Poland). Total genomic DNA was extracted from the leaves of young plants (stage of 3–6 leaves) of the parental lines and the F_1_ and 92 RILs grown in plastic greenhouses using a GeneElute™ Plant Genomics MiniPrep Kit (Sigma-Aldrich, St. Louis, MO, USA) according to the manufacturer’s instructions. PCR amplification was performed using a Mastercycler EP thermocycler (Eppendorf, Hamburg, Germany). The PCR program and reaction mixture were described previously (Słomnicka et al. 2018). For CAPS markers, the reaction mixture for enzymatic digestion contained 10 μL of PCR product, 3.5 μL of sterile water, and 0.1 μL of restriction enzyme (10 U/μL), which were then incubated at 37 °C, 55 °C, or 65 °C for 6 h, respectively, according to the manufacturer’s instructions. PCR products and enzyme-digested products were detected on 3% agarose gels. A total of DArTSeq and SSR markers complemented by CAPS and INDEL markers were used to construct a high-density genetic map of chromosome 6 to narrow the genomic regions corresponding to the identified QTLs.

### RNA isolation and RT–qPCR

Total RNA was isolated from leaf explants of the Gy14 and B10 lines using an RNeasy Plant Mini Kit (QIAGEN, Hilden, Germany). The tissues for the analysis were collected from three representative plants for each time point: before the initiation of regeneration and 7, 14, and 21 days post initiation of regeneration (0, 7, 14, and 21 dpi). RNA was treated with DNaseI using an RNase-Free DNase Set (QIAGEN, Hilden, Germany). The quality and concentration of the RNA samples were monitored using 1% agarose gel electrophoresis and a NanoDrop 2000 Spectrophotometer (Thermo Fisher Scientific, Cleveland, OH, USA). cDNA was synthesized using a Transcriptor High Fidelity cDNA Synthesis Kit (Roche, Basel, Switzerland) according to the manufacturer’s instructions. All RT–qPCR assays were performed using three biological and three technical replicates for each line and time point and three no-template controls (NTCs). The expression study was carried out using a CFX96 Touch Cycler (Bio-Rad Laboratories, Hercules, CA, USA) with Master Mix Maxima SybrGreen qPCR MM 2×ROX (Thermo Fisher Scientific, Cleveland, OH, USA) according to the manufacturer’s instructions. The qPCR program was as follows: 50 °C for 2 min, 95 °C for 10 min, followed by 39 thermal cycles of 15 s at 95 °C and 1 min at 58 °C for candidate genes and at 60 °C for reference genes. Melting curve analysis was performed immediately after qPCR. The temperature range used for the melting curve generation was from 70 to 95 °C. Three genes encoding clathrin adaptor complex subunit (CACS), ubiquitin extension protein (UBI-ep), and TIP41-like family protein (TIP41), which were found to be the most stable in previous studies, were used as references (Słomnicka et al. 2021). Gene-specific primers were used for RT–qPCR (Supplementary Table S2). The relative normalized expression (2^−ΔΔCt^ method) of genes and statistical analysis (Student’s *t*-test) were performed to determine significant differences using CFX Manager v3.1 software (Bio-Rad Laboratories, Hercules, CA, USA).

## Results

### Regeneration efficiency of the cucumber inbred lines and RIL population

**Fig. 1:**
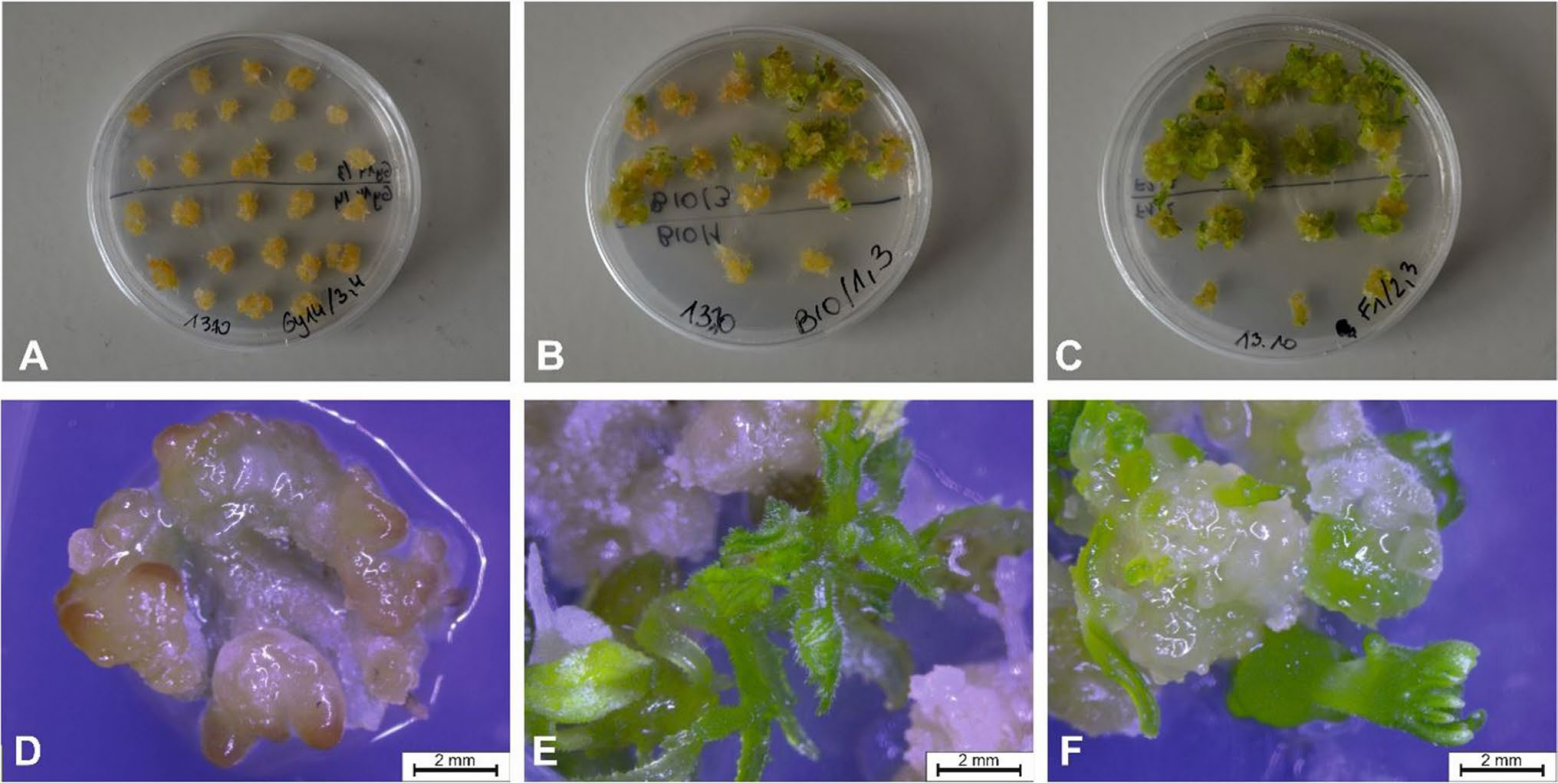
Response of leaf microexplants six weeks after the induction of regeneration for the parental lines Gy14 (**A**, **D**), B10 (**B**, **E**), and F_1_ (**C**, **F**). Bars represent 2 mm

**Fig. 2:**
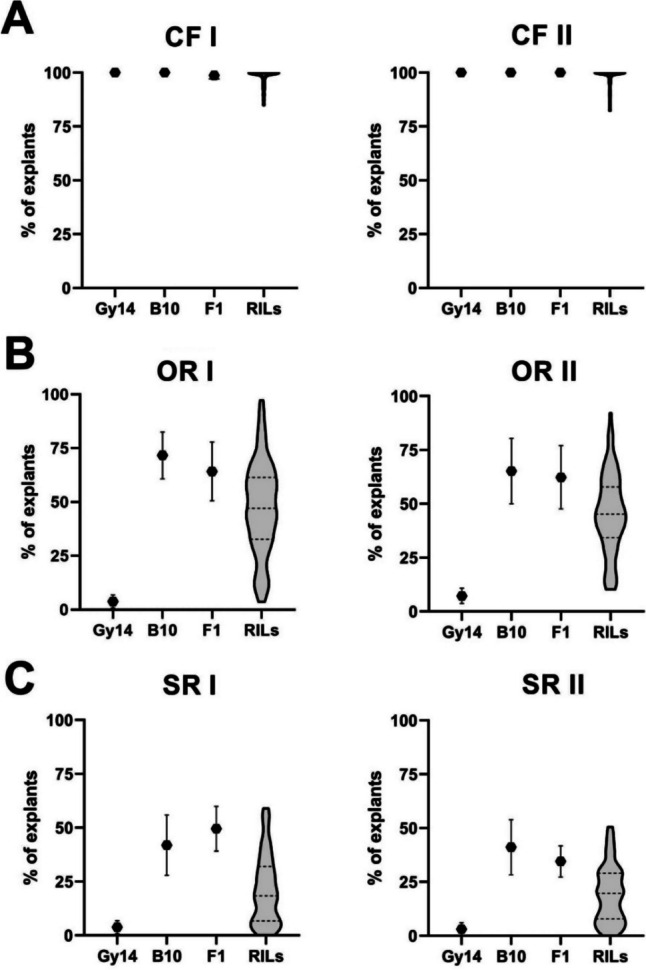
Violin plots representing the distributions of regeneration trait values in parental lines Gy14 and B10, the F_1_ hybrid, and the F_8_ RILs in two independent experiments (I and II). **A** The frequency (%) of explants formed calli (CF I and II). **B** The frequency (%) of explants showing organogenesis (OR I and II). **C** The frequency (%) of explants regenerating shoots (SR I and II)

The frequencies of callus, organ, and shoot formation from leaf microexplants were scored for lines Gy14 and B10 and the F_1_ and F_8_ RILs (Fig. 1). Explants from both the parental lines and F_1_ plants formed calli, the frequency of callus formation 2 weeks after the initiation of regeneration was nearly 100%, and no differences were detected (Table 1, Fig. 2). However, more dynamic and intensive callus formation was observed for line B10 and F_1_ than for line Gy14. The callus formation frequency for 92 RILs in the mapping population ranged from 85 to 100% in experiment 1 and from 82.5 to 100% in experiment 2 (Table 1, Fig. 2).

**Table 1:** Frequencies (%) of explants that formed calli (CFs), exhibited organogenesis (ORs), and regenerated shoots (SRs) from leaf microexplants of the parental lines Gy14 and B10, the F_1_ and F_8_ RILs

Trait	Trial	Parental lines		F_1_	F_8_ RILs		
		Gy14	B10		Mean ± SD	Range	Skewness
CF (callus formation) frequency (%)	I	100.0	100.0	99.2 ± 2.3	98.8 ± 2.9	85.0–100.0	n.a
	II	100.0	100.0	100.0	99.1 ± 2.6	82.5–100.0	n.a.
OR (organogenesis) frequency (%)	I	3.8 ± 3.0 (a)	71.7 ± 10.8 (b)	64.2 ± 13.7 (b)	47.4 ± 20.8	3.6–97.4	-0.04
	II	7.2 ± 3.7 (a)	65.2 ± 15.2 (b)	62.3 ± 14.7 (b)	45.5 ± 18.2	10.3–92.2	0.05
SR (shoot regeneration) frequency (%)	I	3.8 ± 3.0 (a)	41.9 ± 10.0 (b)	49.5 ± 10.4 (b)	21.4 ± 15.8	0.0–59.0	0.64
	II	2.7 ± 3.5 (a)	41.1 ± 12.8 (b)	34.5 ± 7.2 (b)	19.4 ± 13.0	0.0–50.5	0.45

Statistical analyses for OR and SR included one-way ANOVA with Tukey’s post hoc test (*p* < 0.01). Letters in parentheses represent homogeneous groups

*n.a.* not analyzed

Leaf microexplants derived from B10 and F_1_ showed high efficiency of organ regeneration manifested by the formation of roots, leaf-like structures, and buds and shoots, in contrast to explants of Gy14, which formed calli that turned brown over time and only sometimes formed organs (Fig. 1). The frequencies of organogenesis assessed 10 weeks after regeneration initiation were 3.8% and 7.2% for line Gy14 and 71.7% and 65.2% for line B10 in the two independent experiments. The F_1_ demonstrated organogenesis frequency similar to that of the line B10, at 64.2% and 62.3%, respectively (Table 1, Fig. 2). Statistical analysis revealed significant differences (*p* < 0.01) in organogenesis frequency between the lines Gy14 and B10, as well as between Gy14 and the F_1_. Similar observations of shoot regeneration frequency were observed for the parental lines and their F_1_ (Table 1). The frequencies of shoot regeneration were 3.8% and 2.7% for Gy14 and 41.9% and 41.1% for the B10 line across the two experiments. The shoot regeneration frequencies for the microexplants of the F_1_ hybrids were 49.5% and 34.5%, respectively (Table 1). The means differed significantly between the Gy14 and B10 lines and between the Gy14 and F_1_ lines (*p* < 0.01), while the differences between the B10 line and the F_1_ line were not significant (Table 1).

Organogenesis and shoot regeneration frequency among the RILs showed broad and continuous variation in both experiments, ranging from 3.6 to 97.4% and from 10.3 to 92.2%, respectively, for organ regeneration frequency and from 0 to 59% and 0 to 50.5%, respectively, for shoot regeneration frequency. Interestingly, the three RILs exhibited substantial transgressive effects, with greater organ and shoot regeneration frequencies in the parental lines (Table 1, Fig. 2). Therefore, three RILs, lines 523, 637, and 656, were selected to establish a highly efficient regeneration protocol for cucumber (Supplementary Table S3). The normal distribution of organ regeneration frequency among the 92 RILs in both experiments was confirmed by the Shapiro–Wilk test. The distribution of shoot regeneration frequency in both experiments was positively skewed (Table 1, Supplementary Table S4).

### Identification of QTLs for cucumber regeneration efficiency from leaf microexplants

**Fig. 3:**
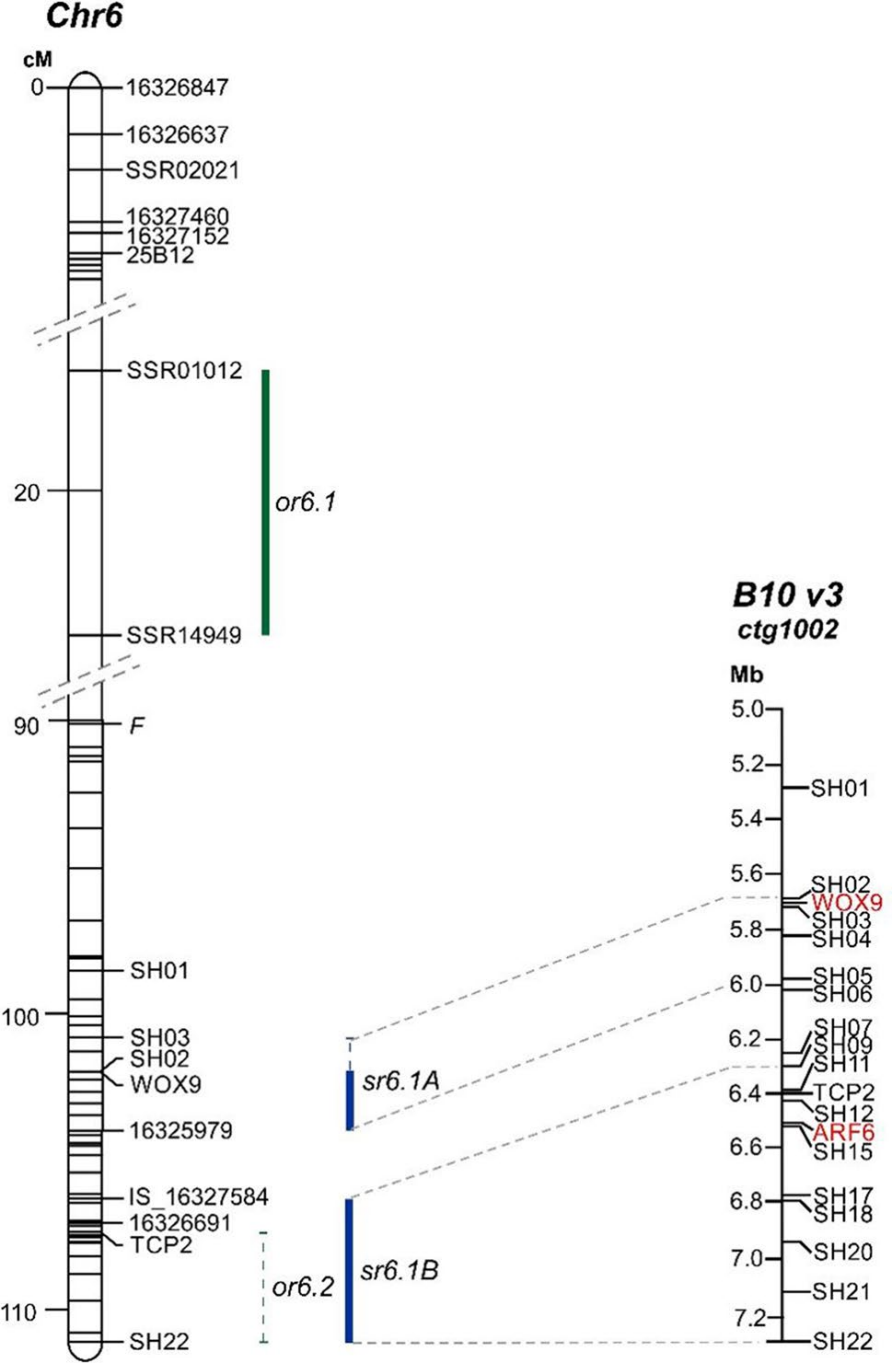
Genetic and physical maps of QTLs for *in vitro* regeneration ability from leaf microexplants in cucumber. QTLs *or6.1* and *or6.2* are associated with organogenesis frequency, and QTLs *sr6.1A* and *sr6.1B* are associated with shoot regeneration frequency. Bars and whiskers represent QTLs found to be significant at *p* ≤ 0.01. Bars represent QTLs identified for both regeneration experiments; whiskers represent QTLs identified for only one regeneration experiment. The green and blue bars denote QTLs for organ and shoot regeneration frequency, respectively. The position of the *Femaleness* locus for gynoecy is marked by *F*. Genes *CsARF6* and *CsWOX9* are in red

A genetic map constructed for the Gy14×B10 RILs population was used to identify QTLs for organogenesis and shoot regeneration frequency. QTL mapping resulted in the identification of two loci for organogenesis and one for shoot regeneration, all of which are located on cucumber chromosome 6. The QTLs for organogenesis frequency were designated as *or6.1* and *or6.2*, and the QTL for shoot regeneration frequency was designated as *sr6.1*. In addition, the frequency of the *sr6.1* QTL for shoot regeneration overlapped with that of *or6.2* for organogenesis. Genomic regions corresponding to *or6.1*, *or6.2*, and *sr6.1* were identified using positions of the markers flanking those loci and were estimated to be 1.2, 0.65, and 1.9 Mb, respectively (Supplementary Fig. S3). To increase the density of the genetic map, a set of CAPS and INDEL markers were developed and tested on the RILs (Supplementary Fig. S2, Supplementary Table S1). Based on the improved genetic map of chromosome 6, *sr6.1* was split into two QTLs, and thus, two QTLs for organogenesis and two for shoot regeneration frequency were identified (Fig. 3, Supplementary Fig. S4, Supplementary Table S5).

On the improved genetic map, QTL *or6.1* was flanked by the markers SSR01012 and SSR14949 at distance ranging from 17.9 to 22.5 cM, and *or6.2* was flanked by the markers TCP2 and SH22 at distance ranging from 107.4 to 111.1 cM (Table 2, Fig. 3, Supplementary Table S5). The LODs for *or6.1* were 3.77 and 3.57, explaining 14.9% and 11.9%, respectively, of the phenotypic variance in the two experiments. The second QTL, *or6.2*, explained 12.1% of the phenotypic variance and was identified only in the second experiment (Table 2). Thus, the identified *or6.1* is a major-effect QTL for organogenesis.

**Table 2:** Characteristics of QTLs controlling cucumber *in vitro* regeneration from leaf microexplants

Trait	QTL	Chr	Trial	Flanking markers	Marker interval (cM)	LOD	PVE% *R*^2^	Additive effect	Number of anchored markers	Genomic region (Mb)
OR frequency (%)	*or6.1*	6	I	SSR01012-SSR14949	17.9–22.5	3.77	14.9	− 0.078	2	1.20
			II	SSR01012-SSR14949	17.9–22.5	3.57	11.9	− 0.063	2	1.20
	*or6.2*	6	II	TCP2-SH22	107.4–111.1	3.61	12.1	0.060	11	0.84
SR frequency (%)	*sr6.1A*	6	I	SH03-16325979	100.8–103.5	3.85	18.1	0.069	8	0.25
			II	SH02-16325979	102.0–103.5	3.82	15.8	0.053	6	0.25
	*sr6.1B*	6	I	IS16327584-SH22	106.3–111.1	4.17	19.4	0.069	18	0.89
			II	16326691-SH22	107.1–111.1	3.66	20.0	0.056	14	0.85

Genomic regions of the QTL established based on cucumber genome B10 v3

*OR* organogenesis, *SR* shoot regeneration, *PVE% (R*^*2*^*)* the percentage of phenotypic variance explained by the QTL

For shoot regeneration frequency, the QTL *sr6.1* was split into two QTLs, designated *sr6.1A* and *sr6.1B*. The QTL *sr6.1A* was mapped between markers SH03 and 16325979 at positions from 100.8 to 103.5 cM. The percentages of phenotypic variance explained by *sr6.1A* were 18.1% and 15.8%, with LODs of 3.85 and 3.82, respectively, in both experiments. The QTL *sr6.1*B was flanked by the markers IS_16327584 and SH22 at positions ranging from 106.3 to 111.1 cM, overlapping with *or6.2* (Table 2, Fig. 3, Supplementary Table S5). The LODs for *sr6.1B* were 4.17 and 3.66, explaining 19.4% and 20%, respectively, of the phenotypic variance in the two experiments. Thus, *sr6.1B* was recognized as a major-effect QTL for shoot regeneration frequency.

Genomic regions corresponding to the QTLs were identified based on the genomic positions of the flanking markers. The interval of QTL *or6.1*, flanked by markers SSR01012 and SSR14949, was estimated to be 1.2 Mb. Based on the genome analysis, 130 annotated genes were identified in this region. This region was similar to the genomic region of the QTLs identified for regeneration ability from cotyledonary explants (Wang et al. 2018). The genomic region corresponding to the major-effect QTL *sr6.1B* was 0.89 Mb long with 113 genes. For the minor-effect *sr6.1A* QTL, the genomic interval corresponded to 0.25 Mb, with 41 annotated genes (Supplementary Table S6). Based on the functional annotation of the genes within the regions corresponding to the major-effect QTL *sr6.1B*, cucumber orthologs of *AUXIN RESPONSE FACTOR 6* (*CsARF6*, Cucsat.G2079) and *TEOSINTE BRANCHED1/CYCLOIDEA/PCF 2* (*CsTCP2*, Cucsat.G2072) were identified as putative candidate genes underlying this QTL*.* Additionally, a cucumber ortholog of *WUSCHEL-related homeobox 9* (*CsWOX9*, Cucsat.G2030) was identified as a putative candidate for QTL *sr6.1A* (Supplementary Table S6). To validate these putative candidate genes, expression profiling at the transcript level was performed.

### Analysis of candidate genes by expression profiling

**Fig. 4:**
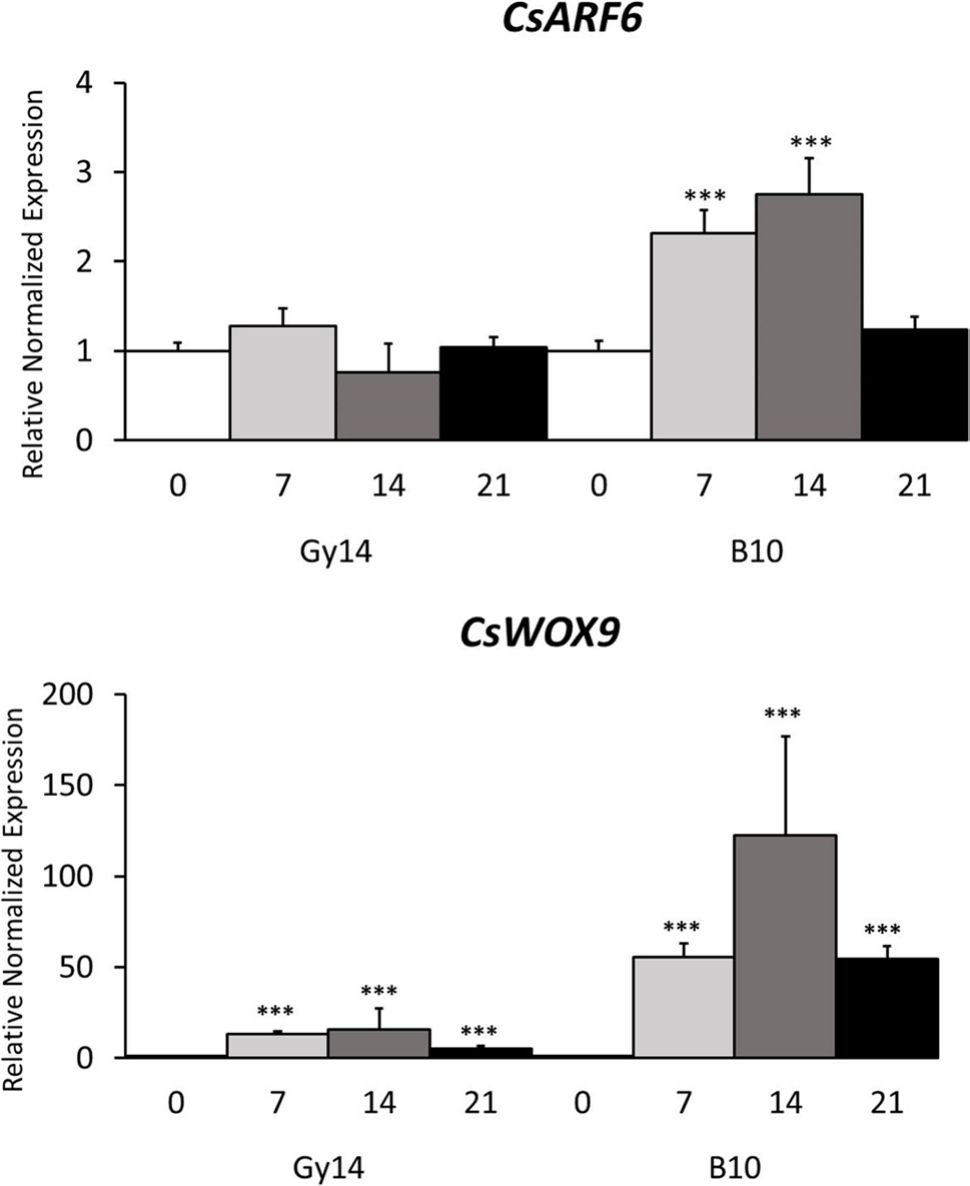
RT–qPCR expression profiles of two candidate genes in lines Gy14 and B10 at 0, 7, 14, and 21 days post initiation of regeneration (dpi). The graphs show the relative normalized gene expression levels ± SEMs for *CsARF6* and *CsWOX9*, as revealed by the analysis of three biological and three technical replicates. Three reference genes, *CACS*, *TIP41*, and *UBI-ep*, were used for data normalization. The complete gene names are provided in Supplementary Table S5. Significance levels were calculated with Student’s *t*-test: *p* < 0.05 (*), *p* < 0.01 (**), and *p* < 0.001 (***)

The expression patterns of *CsARF6*, *CsTCP2*, and *CsWOX9* were examined using RT–qPCR in the parental lines Gy14 and B10 before and 7, 14, and 21 days after the initiation of regeneration (Supplementary Table S7). The expression of *CsARF6* was upregulated in B10 at 7 and 14 days after the initiation of regeneration, and it decreased to a level similar to that before regeneration initiation at 21 dpi. In contrast, in line Gy14, the temporal expression of *CsARF6* did not increase at any point after initiation of regeneration (Fig. 4, Supplementary Table S7). The temporal expression pattern of *CsTCP2* was similar and did not differ between the parental lines (Supplementary Table S7). The expression of *CsWOX9* increased at 7, 14, and 21 days after the initiation of regeneration in both parental lines, but it was significantly higher in B10 than in Gy14 (Fig. 4, Supplementary Table S7). Thus, we concluded that *CsARF6* is a good candidate gene underlying *sr6.1B* and that *CsWOX9* is a candidate underlying *sr6.1A*, two novel QTLs for shoot regeneration efficiency in cucumber identified in this study.

## Discussion

*De novo* *in vitro* plant regeneration may be achieved using two main processes, namely, organogenesis and somatic embryogenesis. In dicots, *de novo* organogenesis is commonly used in biotechnological methods for crop improvement because the collection of plant explants and *in vitro* regeneration protocols are relatively simple and robust (Duclercq et al. 2011). Efficient and stable regeneration procedures are essential for various applications, such as double haploid production, clonal propagation, genetic transformation, and genome editing, in numerous species, including cucumber (Tan et al. 2022).

In this study, we evaluated cucumber *in vitro* plant regeneration from leaf microexplants using a simple, fast, and efficient regeneration protocol established by Burza and Malepszy (1995) (Supplementary Fig. S1), which was successfully used in cucumber biotechnology. We identified novel QTLs controlling the regeneration process. Two QTLs for organ formation were identified: the major-effect QTL *or6.1*, located at the upper arm of chromosome 6, and the minor-effect QTL *or6.2*, located at the end of chromosome 6. For shoot regeneration frequency, also, two QTLs, *sr6.1A* and *sr6.1B*, both at the end of chromosome 6 were identified (Fig. 3). As *or6.2* and *sr6.1B* overlapped, thus, three genomic regions on chromosome 6 were found to be important for plant regeneration from cucumber leaf microexplants. This finding is in agreement with a previous study by Nadolska-Orczyk and Malepszy (1989), in which three interacting genes were shown to control plant regeneration from leaf explants. Furthermore, in a similar study on plant regeneration from cotyledon explants, four QTLs for shoot regeneration on chromosomes 1, 3, and 6, with major-effect QTLs on chromosome 1, were identified (Wang et al. 2018). The QTLs for plant regeneration from cotyledons, described by Wang et al. (2018), explained 9.7 to 16.6% of the phenotypic variance, which is consistent with our results for leaf microexplants, were QTLs controlling regeneration from leaf microexplants explained 11.9 to 20% of the phenotypic variance (Table 2). Thus, based on these studies, it seems that overall three to four major loci control *in vitro* plant regeneration in cucumber.

In the study by Wang et al. (2018), two minor-effect QTLs for shoot regeneration frequency from cotyledons were found on chromosome 6. One of them, *Fcrms+6.2*, was detected on MS+ regeneration medium consistently in two independent experiments, explaining 14.6% and 15.8% of the phenotypic variance, while the second one, *Fcrms6.1/Fcrms*_*+*_*6.1*, was detected on two regeneration media (MS and MS+) but only in single experiments. It appears that the QTLs found on chromosome 6 by Wang et al. (2018) are located in a similar genomic region of *or6.1* identified in this study. We concluded that the QTLs *sr6.1B* and *sr6.1A* are newly identified QTLs that control *in vitro* shoot regeneration from cucumber leaf explants, and we investigated these QTLs in more detail and proposed putative candidate genes.

Although the importance of auxin and cytokinin in plant regenerative pathways has been known since the 1950s, the molecular basis underlying their mechanisms of action has recently been revealed. In *Arabidopsis*, mutants impaired in *in vitro* plant regeneration were identified and used to clone genes involved in organogenic callus formation, shoot organogenesis, and plant regeneration (Cary et al. 2002; Che et al. 2006; Che et al. 2007; Gordon et al. 2007). It has been reported that the *LATERAL ORGAN BOUNDARIES (LOB) DOMAIN* (*LBD*) gene, which encodes a plant-specific transcription factor, is required for callus formation and organ regeneration (Fan et al. 2012; Lee et al. 2019). Transcription factors belonging to the AUXIN RESPONSE FACTOR (ARF) family can increase the expression of *LBDs* to promote cell proliferation and callus formation (Okushima et al. 2007; Ikeuchi et al. 2019). Based on the annotation of the cucumber reference genome, we identified the *CsARF6* gene (Cucsat.G2079), an ortholog of *ARF6*, within the genomic region of the major-effect QTL *sr6.1B*. Within this region, we also identified a gene, *CsTCP2* (Cucsat.G2072), encoding a transcription factor containing a bHLH DNA-binding motif known as the TCP domain (Supplementary Table S6). The transcription factors TEOSINTE BRANCHED1/CYCLOIDEA/PCFs (TCPs) are associated with shoot regeneration (Qiao and Xiang 2013; Yang et al. 2020; Challa et al. 2019)*.* In our study, we examined the expression patterns of *CsARF6* and *CsTCP2*, which are suitable candidates underlying the *sr6.1B* QTL for regeneration efficiency from leaf microexplants. The temporal pattern of *CsARF6* expression significantly differed between the Gy14 and B10 lines, while the expression pattern of *CsTCP2* was similar in both lines (Fig. 4, Supplementary Table S7). Thus, the *CsARF6* gene is a promising candidate underlying the *sr6.1B* QTL for *in vitro* cucumber shoot regeneration from leaf microexplants. In *Arabidopsis*, ARF6/8 are positive regulators of adventitious rooting and are closely related to root organogenesis (Gutierrez et al. 2009). Moreover, *ARF6/8* expression was significantly upregulated in embryogenic cultures of *Arabidopsis* suggesting that these genes are involved in somatic embryogenesis (Wójcikowska and Gaj 2017).

Shoot regeneration from callus requires formation of a primary meristem or a shoot apical meristem (SAM), with a population of pluripotent stem cells, from which the aboveground organs of the plant are differentiated. The WUSCHEL/CLAVATA negative feedback loop plays a major role in maintaining undifferentiated stem cells (Sarkar et al. 2007). Transcription factors belonging to the WUSCHEL-related homeobox gene family (WOX) may be involved in plant growth and development, maintaining meristematic stem cells, embryo development and polarization, lateral organ development, and organ regeneration (Ikeuchi et al. 2019; Gu et al. 2020). WOX9 has been identified as a factor that promotes the growth of the SAM, is required for the maintenance of WUSCHEL expression at the shoot apex, and is involved in cytokinin-mediated signaling during shoot meristem formation (Wu et al. 2005; Skylar et al. 2010). In *Medicago truncatula*, overexpression of *WOX9* improved the efficiency of somatic embryogenesis by increasing the expression levels of two homologs of the *MADS-box* gene, *AGAMOUS*-*like 15 (AGL15)* and *AGAMOUS*-*like 8* (*AGL8*) (Tvorogova et al. 2019). Coexpression of a pair of *WOXs*, such as *WOX2* and *WOX8* or *WOX2* and *WOX9*, was shown to promote plant regeneration from leaf segments in *Nicotiana tobaccum* (Kyo et al. 2018). In this study, in the genomic region corresponding to the *sr6.1A* QTL, we found a cucumber homolog of *CsWOX9* (Cucsat.G2030) (Supplementary Table S6). We showed that the expression of *CsWOX9* was much higher in all the B10 samples after the initiation of regeneration than in the Gy14 samples (Fig. 4). Interestingly, it was reported that WOX9 can directly bind to ARF5 via its C-terminal domain (CTDW) to form the WOX9-ARF5 complex, which initiates primary root formation in *A. thaliana.* Moreover, CTDW differs among WOX proteins and binds specifically to various ARF proteins to form WOX-ARF complexes that play diverse roles in initiating different types of roots (Zhang et al. 2023). Thus, it is possible that in cucumber, the CsWOX9 and CsARF6 interaction is important for inducing organogenesis and shoot regeneration during *in vitro* regeneration. However, further studies are needed to verify this hypothesis.

This study is a step toward identifying QTLs and candidate genes involved in *de novo* plant regeneration from cucumber leaf microexplants. These genes could help to establish highly efficient genetic transformation and gene editing protocols for functional studies and cucumber improvement. Although transformation protocols in cucumber have been continuously improved for many years, transformation systems have only been developed for some cucumber lines (Tan et al. 2022). The efficiency of genetic transformation in cucumber is commonly low, ranging from 1 to 10% (Zhang et al. 2017) which makes the application of gene editing a challenging and daunting task. In monocots, the use of genes encoding function-enabled morphogenic transcription factors (MTFs) such as BABY BOOM, WUSCHEL 2, GROWTH-REGULATING FACTORs (GRFs), and WUSCHEL-homeobox 5 can increase plant transformation efficiency and expand the range of species and genotypes successfully transformed (Lee and Wang 2023). Applying *WUSCHEL 2*–enabled transformation in sorghum increases the transformation efficiency range from 0.7–21% to 20–60% and enhances CRISPR/Cas-targeted genome editing frequency 6.8-fold (Che et al. 2022). The overexpression of the wheat gene *TaWOX5* dramatically increases transformation efficiency with less genotype dependency (Wang et al. 2022a, 2022b). In dicots, MTFs also provided tissue culture-free options for gene editing and transformation by stimulating direct shoot regeneration (Maher et al. 2020). The use of *PLETHORA5*–enabled transformation has been demonstrated to increase transformation efficiency in snapdragon, tomato, cabbage, and sweet pepper (Lian et al. 2022). Thus, this study identified *CsARF6* and *CsWOX9* genes, which can be interesting candidates for developing novel vectors and systems for efficient transformation and gene editing in cucumber.

Moreover, in this study, we identified three RILs (lines 523, 637, and 656) that exhibit substantial transgressive effects, surpassing the organ and shoot regeneration frequencies of the inbred line B10, which is known for its high regeneration efficiency (Supplementary Table S3). Interestingly, all three of these RILs are gynoecious, with white-spined fruits, similar to those of the maternal line Gy14. These three lines provide valuable new resources that can be harnessed for *in vitro* plant regeneration and transformation or gene editing protocols in the future. Overall, this study provides essential insights and new resources for further advancements in cucumber biotechnology, paving the way for more effective strategies for enhancing *in vitro* regeneration and plant transformation in cucumber.

## Supplementary information

ESM 1 Supplementary Table S1. List of primers used to improve the genetic map of chromosome 6. Supplementary Table S2. List of primers used for RT–qPCR. Supplementary Table S3. Phenotypic evaluation of the parental lines Gy14 and B10, the F_1_ hybrid and the F_8_ RILs of the mapping population from Gy14×B10. OR I and OR II - frequencies (%) of explants that formed organs. SR I and SR II- frequencies (%) of explants that formed shoots. Supplementary Table S4. The Shapiro–Wilk test was used to assess the normality of the phenotypic data. Supplementary Table S5. List of loci on the genetic map of chromosome 6. Supplementary Table S6. Annotation of genomic regions corresponding to *or6.1, or6.2, sr6.1A* and *sr6.1B.* Supplementary Table S7. Detailed results of RT–qPCR analysis. Gy14 before induction of regeneration (0 days post-induction, 0 dpi) was used as a control for Gy14 samples at 7, 14, and 21 dpi. Similarly, B10 at 0 dpi was used as a control for B10 samples at 7, 14, and 21 Dpi (XLSX 70 kb)

ESM 2 Supplementary Figure S1. Schematic representation of the *in vitro* regeneration system used in this study. Supplementary Figure S2. Genotyping results of the mapping population B10 × Gy14 using WOX9 as an example of the CAPS marker (A) and SH07 as an example of the INDEL marker (B). Supplementary Figure S3. Chromosome location of QTLs for *in vitro* regeneration in cucumber, *or6.1 and or6.2* for organ regeneration frequency and *sr6.1* for shoot regeneration frequency. Supplementary Figure S4. LOD scores along cucumber chromosome 6 for variation in regeneration traits in two independent experiments. (DOCX 4062 kb)
