# Supplementary material for: Identification of quantitative trait loci for in vitro plant regeneration from leaf microexplants in cucumber (Cucumis sativus L.)
Source: J Appl Genet. 2024 Dec 23;66(3):545–55. doi: 10.1007/s13353-024-00927-3 (PMC12367952; doi:10.1007/s13353-024-00927-3)
Supplement: Supplementary file 2 — Supplementary Figure S1. Schematic representation of the in vitro regeneration system used in this study. Supplementary Figure S2. Genotyping results of the mapping population B10 × Gy14 using WOX9 as an example of the CAPS marker (A) and SH07 as an example of the INDEL marker (B). Supplementary Figure S3. Chromosome location of QTLs for in vitro regeneration in cucumber, or6.1 and or6.2 for organ regeneration frequency and sr6.1 for shoot regeneration frequency. Supplementary Figure S4. LOD scores along cucumber chromosome 6 for variation in regeneration traits in two independent experiments. (DOCX 4062 kb) [file 13353_2024_927_MOESM2_ESM.docx]

# Supplementary Figures





**Supplementary Figure 1.** Schematic representation of the *in vitro* regeneration system used in this study. The *in vitro* regeneration system was used in each of two independent experiments to generate two phenotypic data sets useful for subsequent QTL mapping.


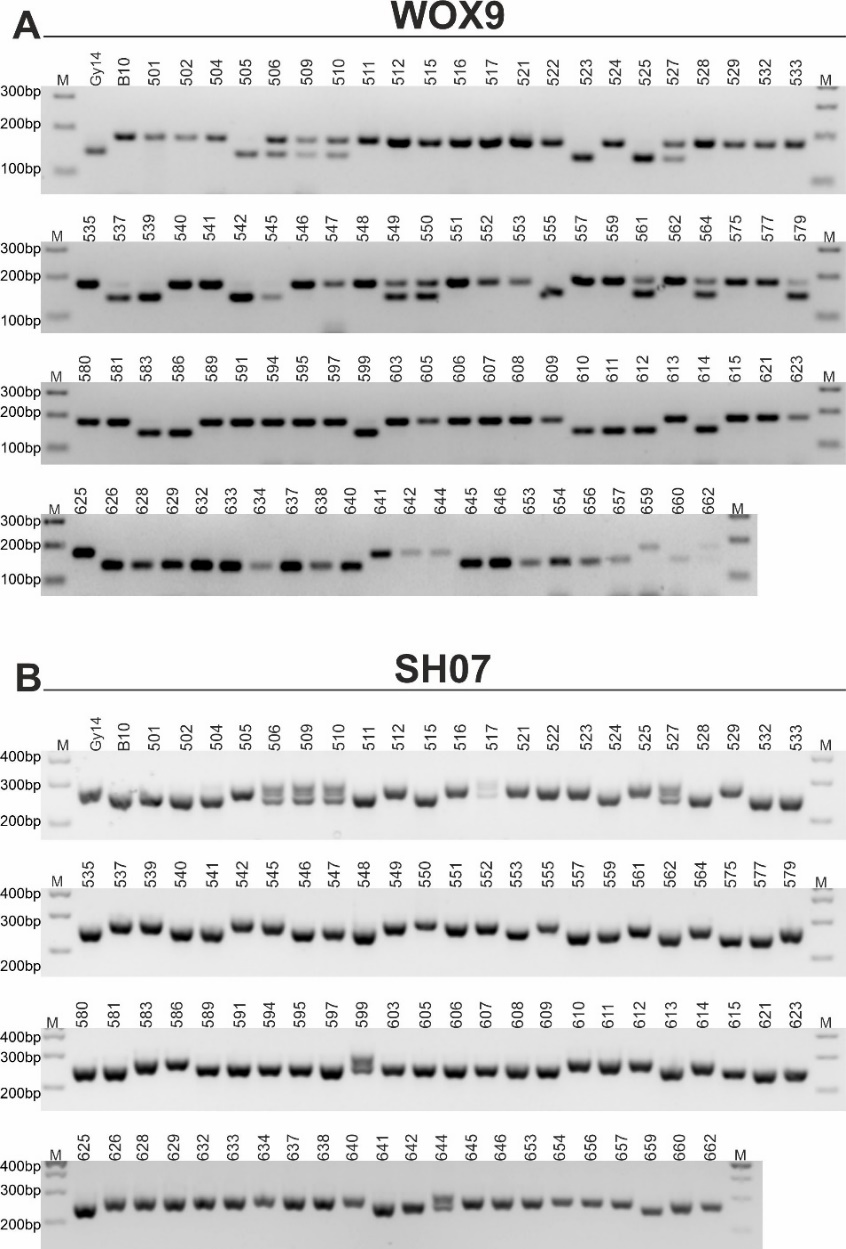


**Supplementary Figure S2.** Genotyping results of the mapping population B10 × Gy14 using WOX9 as an example of the CAPS marker (A) and SH07 as an example of the INDEL marker (B). The PCR product for the WOX9 marker was digested with the HinP1I enzyme. GeneRuler 100 bp Plus DNA Ladder (Thermo Fisher Scientific, Cleveland, OH, USA) was used as the marker (lane M). PCR and enzyme-digested products were detected on 3% agarose gels (0.5×TBE, 120V, 90 min) and ethidium bromide DNA staining.

*
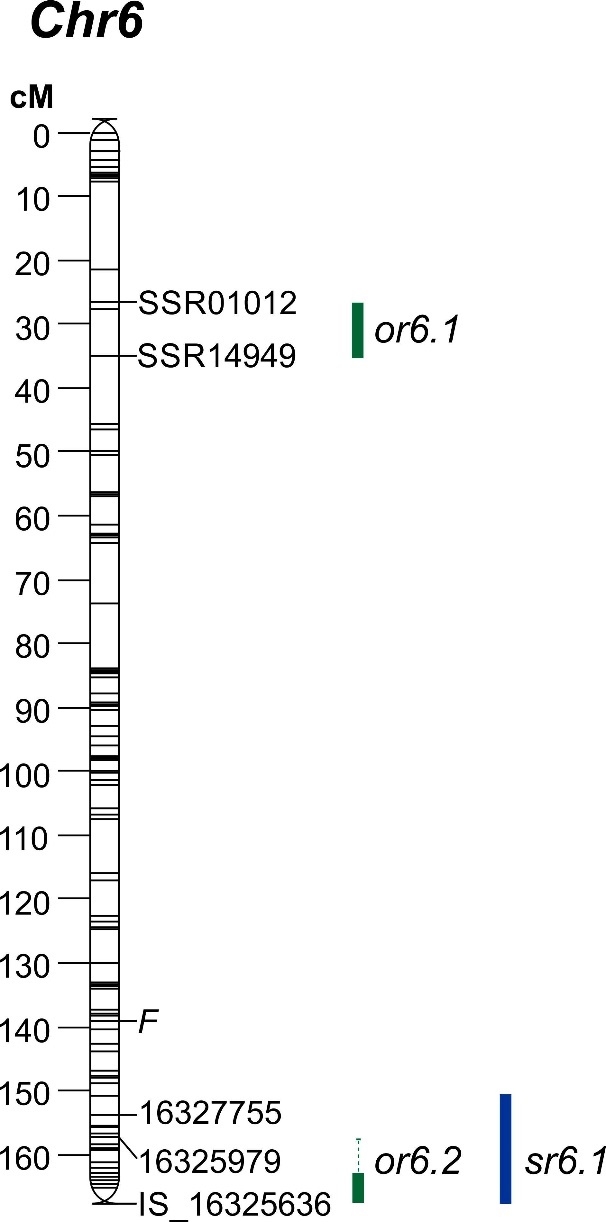
*

**Supplementary Figure S3.** Chromosome location of QTLs for *in vitro* regeneration in cucumber, *or6.1 and or6.2* for organ regeneration frequency and *sr6.1* for shoot regeneration frequency. Bars and whiskers represent QTLs found to be significant at p ≤ 0.05. Bars represent QTLs identified for both regeneration experiments; whiskers represent QTLs identified for only one regeneration experiment. The green and blue bars denote QTLs for organ and shoot regeneration frequency, respectively. The position of the *Femaleness* gene is marked by *F*.

*
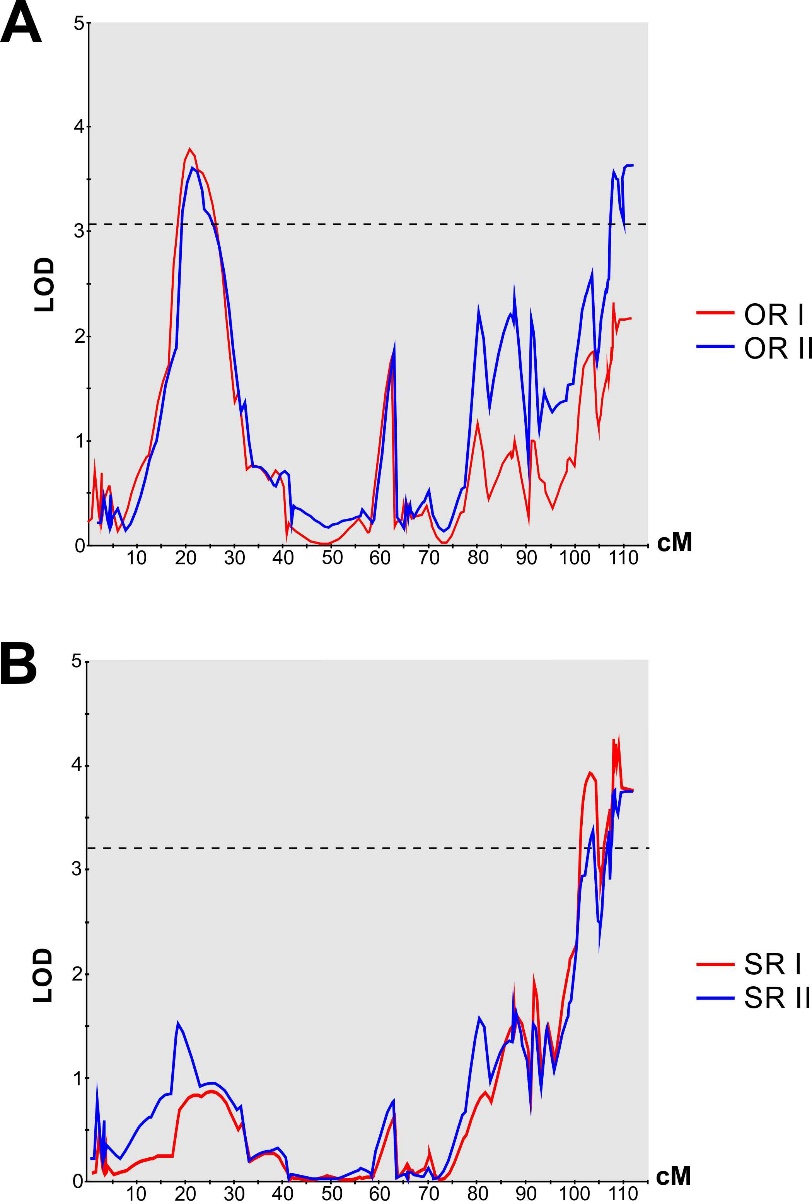
*

**Supplementary Figure S4.** LOD scores along cucumber chromosome 6 for variation in regeneration traits in two independent experiments. (A) QTL mapping for organogenesis frequency (OR I and II). (B) QTL mapping for shoot regeneration frequency (SR I and II). The horizontal dotted line on each trait indicates the LOD for genome-wide significance for p ≤ 0.01. The red line shows the results of the first experiment, and the blue line shows the results of the second experiment.
